# Phase II clinical trial of capecitabine and gemcitabine chemotherapy in patients with metastatic renal carcinoma

**DOI:** 10.1038/sj.bjc.6602209

**Published:** 2004-10-26

**Authors:** J S Waters, C Moss, L Pyle, M James, S Hackett, R A'Hern, M Gore, T Eisen

**Affiliations:** 1 Royal Marsden Hospital, London and Sutton, UK

**Keywords:** renal cell carcinoma, capecitabine, gemcitabine, chemotherapy

## Abstract

We report a single institution phase II study of gemcitabine 1200 mg m^−2^ i.v. on days 1 and 8 and capecitabine 1300 mg m^−2^ twice daily on days 1–14 of each 3-week cycle in patients with metastatic renal carcinoma. Patients had a WHO performance status of 0, 1 or 2. Of the 21 enrolled patients, 19 had received prior immunotherapy or chemoimmunotherapy. All had progressive disease at study entry. In all,19 patients had multiple sites of disease. The median duration of metastatic disease was 12.3 months (range 1.2–78.1 months). Three of the 19 evaluable patients achieved a partial response to treatment, with no complete responses, producing an objective overall response rate of 15.8% (95% CI, 3.4–39.6%). The median time to disease progression was 7.6 months, and median overall survival was 14.2 months. Treatment was reasonably well-tolerated, neutropenia being the most frequently observed grade 3 or 4 toxicity, occurring in 57% of patients. Other side effects were consistent with the established toxicity profile of the two drugs, including diarrhoea, palmar-plantar erythema, fatigue, nausea, vomiting and infection. This combination of gemcitabine and capecitabine has modest activity in immunotherapy-refractory metastatic renal carcinoma with manageable toxicity.

Metastatic renal cell carcinoma has a very poor prognosis with a median survival duration of approximately 9 months. A minority of patients experience a response to immunotherapy with interleukin-2 or interferon-alpha, or a combination of these agents. However, some patients have good quality responses with prolonged disease-free survival, and for this reason immunotherapy has remained the standard first-line treatment for this disease (Medical Research Council Renal Cancer Collaborators, 1999). Combinations of immunotherapy and 5-fluorouracil (5-FU) chemotherapy have produced consistently higher response rates of 20–39% in previously untreated patients, but at the expense of additional toxicity (Atzpodien *et al*, 1995,; Lopez Hanninen *et al*, 1995; Tourani *et al*, 1998; Allen *et al*, 2000; van Herpen *et al*, 2000). As yet there is no randomised evidence supporting the superiority of this approach over standard immunotherapy. Patients failing to respond to immunotherapy have a generally poor outlook. At present, there is no standard therapeutic approach for this group of patients. Single-agent chemotherapy with a variety of drugs has failed to produce response rates much greater than 5% (Yagoda *et al*, 1993). For instance, single-agent gemcitabine produced response rates of 6 and 8.1% in two phase II trials (Mertens *et al*, 1993; De Mulder *et al*, 1996). There have been no published clinical trials of single-agent capecitabine in metastatic renal carcinoma, but single-agent 5-FU has been studied, producing response rates of 5-10% (Yagoda *et al*, 1993; Kish *et al*, 1994).

More interesting activity was observed with combination chemotherapy comprising continuous infusion 5-FU and weekly gemcitabine. This regimen produced a 17% response rate among 41 predominantly immunotherapy or chemotherapy pretreated patients with metastatic renal cell carcinoma in a phase II study undertaken at the University of Chicago Hospitals (Rini *et al*, 2000). This regimen has the drawback of requiring an in-dwelling intravenous catheter together with an ambulatory infusion pump for chemotherapy administration.

Capecitabine is an orally available fluoropyrimidine carbonate activated in a three-step enzymatic conversion to 5-FU. The final step of this conversion is catalysed by the enzyme thymidine phosphorylase, which is overexpressed in tumour cells (Mori *et al*, 2000), thus theoretically providing a degree of specificity for tumour over normal tissue. Capecitabine has replaced 5-FU in chemoimmunotherapy regimens without compromising activity in metastatic renal cell carcinoma (Oevermann *et al*, 2000). The combination of gemcitabine and 5-fluoro-2′-deoxyuridine (an intermediate metabolite of capecitabine) has demonstrated synergistic activity *in vitro* (Ren *et al*, 1998). Several phase I trials have evaluated combinations of gemcitabine and capecitabine, and have shown that full single-agent doses can be delivered in combination (Schilsky *et al*, 2002; Hess *et al*, 2003; Scheithauer *et al*, 2003). This single institution phase II trial aimed to evaluate the activity and toxicity of a combination of gemcitabine and capecitabine in patients with metastatic renal cancer refractory to or unsuitable for immunotherapy.

## PATIENTS AND METHODS

### Protocol population

Patients were eligible for this trial if they had histologically or cytologically verified metastatic renal carcinoma of any histological subtype; one or more clinically or radiologically measurable lesions of at least 1 cm diameter outside previous radiotherapy fields; Eastern Cooperative Oncology Group (ECOG) performance status (PS) 0–2; age greater than or equal to 18 years; life expectancy of at least 12 weeks; and had adequate organ function defined by an absolute neutrophil count of at least 1.5 × 10^9^ l^−1^; platelet count of at least 100 x 10^9^ l^−1^; haemoglobin at least 10g dl^−1^; serum creatinine less than three times the upper normal limit or creatinine clearance at least 50 ml min^−1^; ALT, AST and alkaline phosphatase less than 1.5 times the upper normal limit. Patients were allowed to have received prior treatment for metastatic renal carcinoma including interferon-alpha, interleukin-2, or combined biochemotherapy including bolus 5-FU, but were not allowed to have received gemcitabine, capecitabine or infused delivery of 5-FU previously. Patients were also excluded from entry to this trial if they had not received immunotherapy for metastatic renal carcinoma and were suitable for such treatment, if they had severe or uncontrolled nonmalignant disease, or if they had a history of a previous malignancy likely to interfere with protocol treatment or assessment of outcome. Pregnant or lactating women were also excluded from participation, and all patients with reproductive potential were required to use an effective contraceptive method if they were sexually active. All patients provided written informed consent to participate in the trial, which was approved by the Royal Marsden Hospital Scientific Research and Ethics committees (Protocol Number 2028).

### Study design

This was an open-label, single institution, nonrandomised phase II study. The chemotherapy regimen was administered on an outpatient basis and comprised gemcitabine 1200 mg m^−2^ i.v. over 30 min on days 1 and 8 and oral capecitabine 1300 mg m^−2^ twice daily for 14 days of a 21-day cycle for up to six cycles of treatment. Patients were restaged with physical examination, computed tomography (CT) scan and where applicable radioisotope bone scan after three and six cycles, or in the event of clinical suspicion of disease progression. Treatment was discontinued in the event of disease progression, unacceptable toxicity (including any grade 4 nonhaematological toxicity or symptomatic grade 4 haematological toxicity), or at the patient's request. In the event of grade 3 nonhaematological toxicity other than palmar-plantar syndrome, treatment was stopped and the next cycle delayed until recovery to baseline level. Treatment was then commenced with a 25–50% reduction in gemcitabine and/or capecitabine doses at the investigator's discretion.

In the case of palmar plantar syndrome, treatment was stopped until recovery, and patients were commenced on pyridoxine 50 mg three times a day. For subsequent cycles, no adjustment to the gemcitabine dose was made. For grade 2 toxicity or the first appearance of grade 3 toxicity, the capecitabine dose was reduced by 25%. For grade 4 toxicity or recurrent grade 3 toxicity the capecitabine dose was reduced by 50%.

Delays and dose reductions were also instituted in the event of haematological toxicity as follows. Day 1 gemcitabine and capecitabine treatment was delayed for 1 week if the total white cell count was less than 3.0 × 10^9^ l^−1^ or the platelet count was less than 100 × 10^9^ l^−1^. Day 8 gemcitabine treatment was dose-reduced by 25% if the total white cell count was in the range 2.0–2.5 × 10^9^ l^−1^ or the platelet count was in the range 50–75 × 10^9^ l^−1^. A total white cell count or platelet count below these ranges required omission of the Day 8 gemcitabine, and doses of gemcitabine on subsequent cycles were administered with a 25% dose-reduction. In the event of a delay in treatment due to toxicity of greater than 3 weeks, the patient was removed from the study.

### Response assessment

A complete response was defined as the complete disappearance of all evidence of disease determined by two observations not less than 4 weeks apart. A partial response was defined as a decrease of 50% or more in the sum of the products of the two maximum perpendicular diameters of measurable disease for at least 4 weeks, without the appearance of new lesions, or progression of any individual lesion. Progressive disease was defined as a 25% or more increase in the sum of the products of the two maximum perpendicular diameters of measurable lesions, or the development of any new lesions. Stable disease was defined as any change less than that satisfying the criteria for complete response, partial response or progressive disease over a period of at least 4 weeks. No external verification of response was employed. Time to disease progression was measured from the date of study enrolment to the date of progressive disease. Duration of response was measured from the date objective measurement criteria were met for complete or partial response to the date of disease progression. Overall survival was measured from the date of study enrolment to the date of death from any cause.

### Statistical considerations

The primary end point of this phase II trial was to determine the response rate of gemcitabine and capecitabine in a population of patients with metastatic renal cell carcinoma refractory to or unsuitable for treatment with immunotherapy. The reported response rate to the combination of gemcitabine and 5-FU in this setting was 17% (95% CI, 8–34%) (Rini *et al*, 2000), while no other second-line agent has consistently produced a response rate greater than 10%. This trial adopted a two-stage Gehan design (Gehan, 1979), enrolling 14 patients in the first stage. The treatment would be rejected as insufficiently active if no responses were observed in this stage. In the event of 1, 2, 3, 4, or 5 or more responses among the first 14 patients, 1, 4, 6, 9, or 11 patients, respectively, would be entered in the second stage. This design has a less than 5% probability of rejecting a treatment with a true response rate of 20%, and provides an estimate of the response rate with a standard error of approximately 10%.

## RESULTS

### Patient characteristics

This study accrued patients between March 2002 and March 2003. Three responses were observed during the first phase, leading to the recruitment of an additional six patients according to the trial design. One patient was removed from study due to an incidental chest infection unrelated to study medication before completion of the first cycle. This patient was replaced resulting in 21 patients being enrolled in all. The baseline demographic and clinical characteristics of the patient population are listed in Table 1

**Table 1:** Baseline demographic and disease characteristics

Number of patients enrolled	21
Median age (range)	57 (36–2) years
*Sex*	
Male	15
Female	6
*Performance status*	
0	5
1	13
2	3
*Histology*	
Clear cell	11
Mixed clear cell and granular cell	2
Granular cell	2
Papillary	4
Oncocytic	1
Transitional cell	1
*Prior therapy*	
Nephrectomy	20
Chemoimmunotherapy	5
Immunotherapy	14
Radiotherapy	5
Median number of prior systemic therapies (range)	1 (0–3)
*Involved sites of disease*	
Lung	13
Liver	8
Lymph nodes	11
Bone	7
Renal bed	6
CNS	1^a^
Median number of sites involved (range)	2 (1–5)
Median time since diagnosis of metastatic disease in months (range)	12.3 (1.2–78.1)
Median time since diagnosis in months (range)	18.2 (4.3–297.2)
Number of poor prognostic factors^b^	
0	6
1	9
2	5
3	1

a Brain metastasis previously resected.

b Motzer *et al* (2004).

. The majority of patients had received one prior treatment regimen, comprising either single agent interferon or a chemoimmunotherapy regimen incorporating bolus 5-FU, interferon and IL-2. However, five patients had received two prior treatment regimens, and one patient had received three prior treatment regimens. Only two patients had received no prior treatment for metastatic renal carcinoma: one had previously undergone cadaveric renal transplantation and was considered unsuitable for immunotherapy; the second had a diagnosis of transitional cell carcinoma of the renal pelvis for which immunotherapy is not an appropriate treatment. In view of the different natural history and chemosensitivity of transitional cell carcinoma and epithelial renal cell carcinoma, this patient who achieved a partial response to therapy is considered a protocol violation. We report toxicity data relating to this patient, but not outcome data. The median time since diagnosis of metastatic disease of 12.3 months suggests a patient population that may have relatively indolent disease. However, 38% of patients enrolled had liver metastases, while only one patient had metastatic disease confined to the lungs. Furthermore, considering the prognostic index developed by Motzer *et al*, 2004, incorporating the recognised adverse prognostic factors of poor performance status, anaemia, and hypercalcaemia, 43% of this patient cohort fall into the intermediate and 29% into the poor prognosis categories.

### Response to treatment

One patient was not assessable for response due to the development of a severe chest infection during the first cycle of treatment in the absence of neutropenia. This delayed resumption of chemotherapy beyond the protocol-stipulated limits, and the patient was removed from study. A second patient died after a single dose of gemcitabine, without evidence of chemotherapy-related toxicity. This patient was also not assessable for response, but was included in the intent-to-treat analysis, and was classified as having progressive disease. Among the 19 patients assessed for response, there were no complete responses, and three partial responses (objective response rate, 15.8% (95% CI, 3.4–39.6%). In all, 10 patients (53%) had stable disease, and six patients (32%) experienced disease progression on treatment. Among the three responding patients, the response duration was 5.6, 5.8 and 18.8 months, respectively. The clinical characteristics of these three patients are listed in Table 2

**Table 2:** Characteristics of responding patients

Patient no.	6	8	12
Sex	Male	Female	Male
Age	46	55	44
Prior nephrectomy	Yes	Yes	Yes
Histology	Mixed clear cell and granular	Granular	Granular
Prior systemic therapy	Atzpodien regimen^a^	1. 5-FU + IL-2	Atzpodien regimen^a^
		2. IL-2 + IFN	
Time since diagnosis of metastases (months)	12	13	7
Metastatic sites	Lung	Lung, lymph nodes, bone	Lung, liver
Performance status	0	1	1
Number of poor prognostic factors	1	0	1
Duration of response (months)	5.6	5.8	18.8^b^
Time to disease progression (months)	7.9	7.6	20.8^b^
Post-trial therapy	BAY 43-9006^c^	Provera	None
Overall survival (months)	21.5^d^	9.9	20.8^d^

a IL-2, IFN-alpha and 5-FU (Atzpodien *et al*, 2001).

b Remains free of disease progression on follow-up.

c Phase II trial of a B-raf kinase inhibitor.

d Remains alive on follow-up.

. Of interest, responses were observed in three of four patients with granular cell histology (including one patient with mixed clear cell and granular cell histology), whereas none of the four patients with papillary histology responded to this treatment. The prognostic category of one responding patient was favourable (no adverse risk factors), and of the remaining two was intermediate (one adverse risk factor in each case) (Motzer *et al*, 2004).

**Figure 1:**
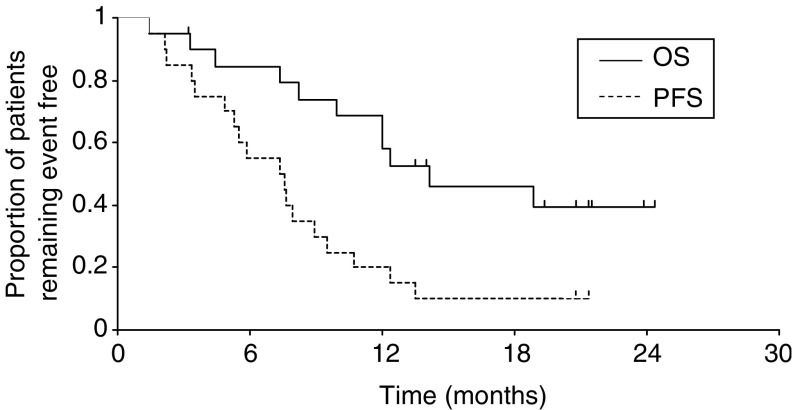
Progression-free and overall survival (*n* = 20).

Kaplan – Meier curves for progression-free and overall survival are shown in Figure 1

. With a median follow-up duration for surviving patients of 20.8 months, two patients remain free of disease progression and the median progression-free survival is 7.6 months. In all, 11 patients have died. The median overall survival duration is 14.2 months, and the estimated proportion of patients remaining alive at 1 year is 58%.

### Toxicity

Toxicity data have been analysed for all 21 patients enrolled in the study, who received a total of 89 cycles of therapy. Overall worst grade of haematological toxicity was grade 0 in one patient, grade 2 in seven patients, grade 3 in nine patients and grade 4 in four patients. Overall worst grade of nonhaematological toxicity was grade 2 in six patients, grade 3 in 12 patients and grade 4 in three patients. The rate of grade 2–4 toxicity is shown in Table 3

**Table 3:** Adverse event

	**Grade 2**		**Grade 3**		**Grade 4**	
**Toxicity**	**No (%) patients**	**% cycles**	**No (%) patients**	**% cycles**	**No (%) patients**	**% cycles**
Neutropenia	4 (19)	23	9 (43)	20	3 (14)	5
Thrombocytopenia	0	2	4 (19)	6	1 (5)	1
Anaemia	10 (38)	14	2 (10)	2	0	0
Infection	6 (29)	16	7 (33)	9	0	0
Palmar-plantar erythema	5 (24)	9	4 (19)	5	0	0
Diarrhoea	2 (10)	2	3 (14)	5	0	0
Nausea and vomiting	6 (29)	7	1 (5)	1	0	0
Lethargy	5 (24)	8	2 (10)	4	0	0
Thromboembolism	0	0	1 (5)	1	2 (10)	2
Skin rash	4 (19)	6	1 (5)	2	1 (5)	1
Haemorrhage	2 (10)	4	1 (5)	1	0	0
Dyspnoea	6 (29)	12	1 (5)	1	0	0
Mood alteration	0	1	1 (5)	1	0	0
Renal impairment	1 (5)	1	2 (10)	2	0	0
Hepatic dysfunction	1 (5)	2	1 (5)	1	0	0

. The most frequently observed grade 3 or 4 toxic effects were neutropenia in 57% of patients, infection in 33% (neutropenic sepsis in 14%), thrombocytopenia in 24%, palmar plantar erythema in 19%, diarrhoea in 14% and thromboembolism in 14%. Despite these relatively high frequencies of severe toxicity per patient, adherence to the protocol-stipulated treatment delays and dose reductions resulted in low rates of severe toxicity per chemotherapy cycle. The most frequently observed grade 3 or 4 toxicity per cycle was neutropenia, occurring in 24% of cycles. Grade 3 or 4 infection was recorded in only 9% of cycles (neutropenic sepsis in 3% of cycles), thrombocytopenia in 7% of cycles, and all other grade 3 or 4 toxicity was observed in fewer than 5% of cycles. Clinical sequelae resulting from myelosuppression included three cases of grade 3 infection in association with grade 3 or 4 neutropenia. Three cases of haemorrhage in association with grade 3 or 4 thrombocytopenia were also observed. However, blood transfusion was only required in one case, and the relationship between the episode of haemorrhage (epistaxis) and the development of anaemia requiring blood transfusion was not established.

Three cases of venous thromboembolism occurred during protocol therapy. Two cases were uncomplicated deep vein thrombosis, and the third was a case of pulmonary embolism. All were treated successfully with standard anticoagulation and were able to continue chemotherapy on study, although frequent monitoring of the INR was required due to the metabolic interaction between capecitabine and warfarin. One patient died during protocol treatment, after a single dose of gemcitabine. There was no evidence of treatment-related toxicity in this case, but a post-mortem examination was not performed. Four patients were removed from study because of toxicity. One patient had prolonged grade 3 or 4 neutropenia and thrombocytopenia, and recurrent diarrhoea despite dose reduction. One patient had recurrent grade 3 palmar plantar erythema despite dose reduction. One patient developed ischaemic chest pain, attributed to capecitabine therapy, and the final patient developed prolonged pyrexia of unknown origin, without microbiological evidence of infection.

### Dose intensity

**Figure 2:**
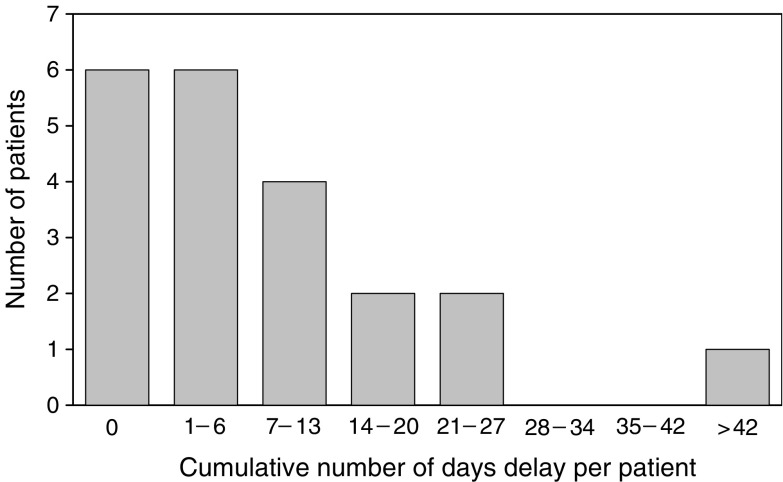
Delays to chemotherapy delivery.

**Figure 3:**
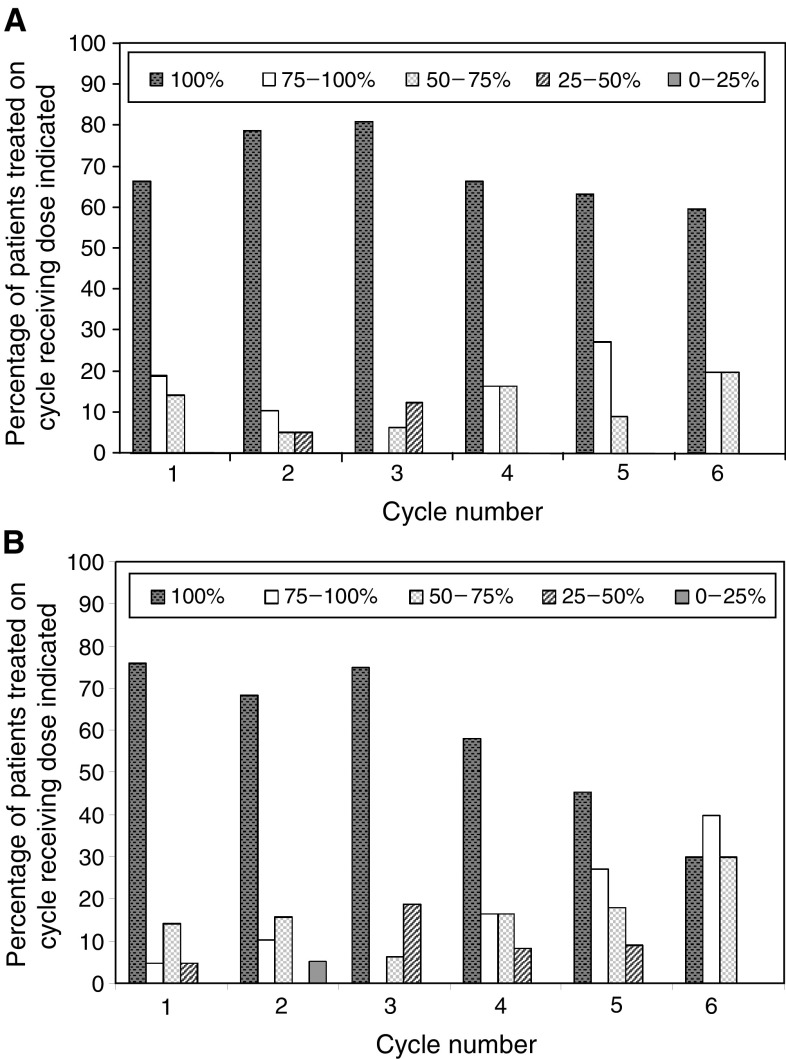
Chemotherapy dose reductions. Bars indicate the percentage of patients who received chemotherapy cycles 1–6 at 100, 75–99, 50–74, 25–49, and <25%, respectively, of planned doses. (**A**) Gemcitabine (**B**) Capecitabine.

Treatment interruptions and dose reductions were frequently required, only two patients completing six cycles of treatment without any modification in dose or schedule. Overall, 72% of cycles were delivered on schedule, and 52% of cycles were delivered at full dose, while 38% of cycles were delivered on schedule and at full dose. The total number of days delay per patient is shown in Figure 2,

and the doses of capecitabine and gemcitabine delivered per cycle are shown in Figure 3

. Using the formula: dose intensity = (cumulative dose received/duration of treatment)/(planned cumulative dose/planned duration of treatment), the achieved dose intensity for gemcitabine was 0.8, and for capecitabine was 0.76. Taking into account cycles of chemotherapy omitted after discontinuation of therapy due to toxicity, achieved dose intensities were 0.7 for gemcitabine and 0.66 for capecitabine.

## DISCUSSION

This phase II trial has demonstrated modest activity of the combination of gemcitabine and capecitabine in a population of patients with immunotherapy-refractory metastatic renal carcinoma. While comparisons between uncontrolled phase II trials must be undertaken with caution, particularly in a disease with a variable natural history such as renal cancer, the response rate of 15.8% observed in patients with epithelial renal cell carcinoma in this trial is comparable to that produced by the combination of gemcitabine and protracted venous infusion 5-FU in the University of Chicago study (Rini *et al*, 2000), and is among the highest reported for any treatment regimen in this patient population. Similarly, the median time to disease progression of 7.6 months is clinically meaningful. The median overall survival duration of 14.2 months is longer than expected for this patient population, and may reflect patient selection for a phase II trial or a contribution of subsequent treatment as well as activity of the regimen under evaluation.

This chemotherapy regimen was generally well tolerated, with little unexpected toxicity. The largely nonoverlapping toxicity profile of the two drugs allowed the delivery of full single-agent doses of capecitabine and gemcitabine in combination. The most common toxic effect was myelosuppression, and this toxicity was the main reason for treatment interruption and dose reduction. The planned dose intensity of gemcitabine in this study was 833 mg m^−2^ week^−1^ in contrast to the University of Chicago study of gemcitabine and 5-FU where the planned dose intensity of gemcitabine was 600 mg m^−2^ week^−1^. This difference probably accounts for the higher rates of myelosuppression observed in our study. However, despite dose reductions and delays, the achieved dose intensity of gemcitabine was 666 mg m^−2^ week^−1^. Clinical sequelae of myelosuppression were infrequent and manageable in all cases, only one patient requiring removal from study because of persistent neutropenia and thrombocytopenia. The 14% incidence of venous thromboembolism observed during this study is not unexpected in this patient cohort. A previous phase II trial of gemcitabine, 5-FU and thalidomide in patients with metastatic renal carcinoma reported a 48% rate of venous thromboembolic complications, which the authors attributed largely to the addition of thalidomide to the regimen (Desai *et al*, 2002).

On the basis of this trial, the combination of gemcitabine and capecitabine is a reasonable treatment option for patients with metastatic renal carcinoma following the failure of standard immunotherapy. However, results of chemotherapy in this group as a whole remain disappointing. There is a suggestion from our results that the granular-cell subtype of renal carcinoma may be more sensitive to gemcitabine and capecitabine chemotherapy than other histological types. This is in contrast with other reports suggesting greater chemosensitivity of papillary renal carcinoma to topotecan (Ramp *et al*, 2001); in our study, none of four patients with this histological subtype achieved a response to treatment. It will clearly be important to attempt to understand fundamental differences between these diseases, which may facilitate the rational selection of therapy for these patients. These observations support the concept of targeted cytotoxic therapy for renal carcinoma. A phase II trial examining the multitargeted antifolate pemetrexed in the treatment of metastatic renal carcinoma demonstrated a response rate of 9% (Thodtmann *et al*, 2003). The combination of pemetrexed and gemcitabine would be of interest for further study. In our view, such trials should incorporate assessment of the expression of key target enzymes such as thymidylate synthase, dihydropyrimidine dehydrogenase and glycinamide ribonucleotide formyl transferase (GARFT).
